# RelA-mediated (p)ppGpp accumulation links glucose starvation to peptidoglycan synthesis arrest and spherical morphogenesis via PBP2x and FtsX in *Streptococcus suis*

**DOI:** 10.1128/jb.00030-26

**Published:** 2026-07-14

**Authors:** Tingting Jiang, Hongyu Wu, Ying Sun, Hengfei Yan, Minghui Ni, Shaohui Li, Menglei Liang, Lelin Zhao, Ziqian Xu, Shuaiyang Wang, Liangsheng Zhang, Lu Li, Rui Zhou, Qi Huang

**Affiliations:** 1 National Key Laboratory of Agricultural Microbiology, Hubei Hongshan Laboratory, College of Veterinary Medicine, Huazhong Agricultural University 47895, Wuhan, Hubei, China; 2 International Research Center for Animal Disease, Ministry of Science and Technology of China, Wuhan, China; 3 The Cooperative Innovation Center for Sustainable Pig Production, Wuhan, China; Indian Institute of Technology Bombay, Mumbai, Maharashtra, India

**Keywords:** (p)ppGpp, *Streptococcus suis*, carbon starvation, cell division, peptidoglycan synthesis

## Abstract

Carbon starvation links nutrient sensing to bacterial morphogenesis, but the molecular pathway connecting glucose limitation to division failure remains unclear. In *Streptococcus suis* SC19, glucose limitation elevated RelA abundance and intracellular (p)ppGpp levels, slowed bacterial growth, and induced a reproducible spherical morphotype associated with selective impairment of peptidoglycan insertion. Inducible and constitutive RelA expression recapitulated the starvation phenotypes and (p)ppGpp accumulation in a dose- and time-dependent manner. The spherical morphotype resulting from glucose starvation-induced RelA activation and consequent (p)ppGpp accumulation coincided with decreased *pbp2x* and *ftsX* transcript levels, whereas *pbp2b*, *ftsW*, *divIVA*, *ftsE*, *pcsB,* and *mltG* displayed divergent expression patterns between starvation and RelA-induction conditions, findings that are consistent with *pbp2x* and *ftsX* acting as principal downstream effectors of the starvation-induced morphotype. Deletion of *ftsX* produced the rounded phenotype, and subinhibitory cefotaxime treatment targeting PBP2x induced progressive rounding; these manipulations together provide functional evidence that FtsX and PBP2x could contribute to (p)ppGpp-dependent PG arrest and spherical morphogenesis. Cross-species validation in *S. pneumoniae* and *E. coli* upon RelA induction produced comparable morphological and transcriptional changes. These results suggest that RelA-mediated (p)ppGpp accumulation couples glucose starvation to PG arrest and spherical morphogenesis via *pbp2x* and *ftsX*, nominating the (p)ppGpp-mediated stringent response as a regulatory hub for peptidoglycan biogenesis during cell division.

**IMPORTANCE:**

Bacterial survival depends on adapting morphogenesis to nutrient availability, yet the molecular links between carbon starvation and cell division remain poorly defined. In the zoonotic pathogen *Streptococcus suis*, glucose limitation triggers RelA-mediated accumulation of the alarmone (p)ppGpp, which arrests peptidoglycan synthesis and induces the morphological transition from ovoid to spherical cells. The downregulation of the key division components PBP2x and FtsX is likely an important driver of this morphological transition. Furthermore, this (p)ppGpp-dependent division “brake” is conserved in *Streptococcus pneumoniae* and *Escherichia coli*. By identifying a direct pathway coupling metabolic status to the division machinery, this work provides fundamental insights into how bacteria preserve cellular integrity under stress, highlighting potential targets for disrupting bacterial persistence and adaptation during infection.

## INTRODUCTION

Bacterial populations commonly remodel their cell envelope and overall morphology in response to nutrient and energy limitation, adopting morphological changes or entering metabolically quiescent states that can include viable-but-non-culturable (VBNC) phenotypes (1). Such shape transitions are widely observed across taxonomic groups and environmental contexts, including zoonotic agents such as *Streptococcus suis* and enteric bacteria like *Escherichia coli*, as well as commensal and soil species, indicating that morphological plasticity is a general adaptive strategy rather than an idiosyncratic response (2). Morphological remodeling can confer physiological advantages under nutrient scarcity by changing surface-to-volume relationships, lowering per-cell maintenance costs, and altering interactions with the microenvironment. Altered cell shapes can improve survival and stress tolerance when resources are limiting (3). Despite these well-documented phenotypes, the molecular pathways that translate specific energetic cues, particularly carbon limitation, into coordinated and reversible programs of envelope remodeling remain unclear (4).

Nutrient limitation, particularly glucose scarcity, rapidly triggers bacterial stringent response, whose biochemical hallmark is intracellular accumulation of the alarmone (p)ppGpp (5). RelA/SpoT-homolog (RSH) enzymes are the principal catalysts of (p)ppGpp turnover. Ribosome-associated *relA*-type synthetases are activated by deacylated tRNA at the A site to synthesize (p)ppGpp (6), while hydrolase domains of RSH proteins and dedicated alarmone-degrading enzymes counterbalance synthesis to establish species-specific alarmone homeostasis (7). Small alarmone synthetases (SASs) provide additional, stress-specific sources of (p)ppGpp in many Gram-positive organisms, permitting rapid and context-dependent modulation of alarmone levels (8). Elevated (p)ppGpp reprograms transcriptional and translational priorities and redirects cellular resources from growth-promoting processes toward stress-survival pathways (9). These properties make (p)ppGpp a plausible molecular link by which carbon limitation can reshape envelope biogenesis and thereby influence cell morphology.

Peptidoglycan (PG) biosynthesis at the division septum is indispensable for bacterial cytokinesis. Coordinated and localized insertion of septal PG builds the cross-wall that separates daughter cells and, thus, determines cell shape during division (10). This process depends on the concerted activity of synthases, including class B transpeptidases, such as PBP2x and their accessory partners, to polymerize glycan strands and form peptide cross-links, together with tightly regulated PG hydrolases that enable controlled septal cleavage and daughter-cell separation (10). Structural and functional studies identify the membrane-associated FtsX as a conserved regulator that couples cytoplasmic division cues to periplasmic amidase activation and gates septal hydrolysis in an ATP-dependent manner (11). Perturbation of septal peptidoglycan synthesis or misregulation of hydrolase activation, for example, by altering PBP abundance or the FtsEX-PcsB system, rapidly arrests cytokinesis and, in ovococcal bacteria, induces spherical cell formation and aberrant septation (12, 13).

Given that (p)ppGpp rapidly reprograms transcription, translation, and enzyme activity under carbon limitation, it can serve as a plausible upstream effector of morphology (14, 15). However, the mechanistic links between (p)ppGpp dynamics and cell-wall assembly are not uniform across species. In some organisms, (p)ppGpp-driven transcriptional changes coincide with altered expression of cell-wall enzymes, whereas in others, ppGpp-dependent morphological outcomes occur without detectable changes in PG composition or steady-state transcripts, implying indirect or species-specific mechanisms (16, 17).

Understanding these mechanisms is particularly critical for zoonotic pathogens that rely on stress responses for persistence and transmission. *Streptococcus suis* (*S. suis*) is a major zoonotic pathogen that inflicts substantial animal-health and economic burdens through meningitis, septicemia, arthritis, and reduced herd productivity; outbreaks and high carriage rates in intensive pig production systems lead to increased mortality, treatment costs, and measurable production losses (18). The pathogen’s persistence and the difficulty of eradication are, in part, attributable to its capacity to mount adaptive stress responses, notably the (p)ppGpp-mediated stringent response, that promote survival under environmental and antimicrobial stress and modulate virulence-associated traits (19). Work from our group has characterized the functions of (p)ppGpp synthetases RelA and RelQ in adaptation to glucose starvation in *S. suis* (20) in which loss of RelA/RelQ attenuates virulence and alters morphology and stress resistance (21), and CodY interacts with RelA to co-regulate morphology and pathogenesis in complex ways (22). These studies demonstrate that (p)ppGpp substantially shapes *S. suis* physiology and pathogenic potential, but they stop short of defining whether and by what molecular mechanisms (p)ppGpp links carbon limitation to PG biogenesis and spherical morphogenesis under starvation. Clarifying this link has clear implications for understanding *S. suis* persistence and for identifying potential intervention points to reduce disease burden.

Here, the hypothesis that (p)ppGpp links carbon limitation to cell-shape remodeling by modulating PG biogenesis and division is tested, particularly septal PG. In *Streptococcus suis* SC19, glucose restriction elevates RelA and intracellular (p)ppGpp, slows growth, and arrests PG insertion, and inducible RelA overexpression reproduces these phenotypes. Transcriptional profiling revealed decreased *pbp2x* and *ftsX* under starvation and RelA induction, whereas *pbp2b*, *ftsW*, *divIVA*, *ftsE*, *pcsB,* and *mltG* showed divergent patterns, results consistent with *pbp2x*/*ftsX* potentially acting as key downstream effectors. Complementary functional perturbations (Δ*ftsX* and subinhibitory cefotaxime at 1/8-1/4 MIC) phenocopy cell rounding, supporting that FtsX and PBP2x could contribute to (p)ppGpp-dependent septal PG arrest. Cross-species tests in *E. coli* and *S. pneumoniae* produced comparable morphological and transcript-level changes. Collectively, these data suggest that (p)ppGpp-associated inhibition of PG biogenesis is a reproducible mechanism driving spherical morphogenesis and nominate septal biogenesis as a tractable axis for future efforts to limit persistence and virulence.

## RESULTS

### Glucose starvation induces spherical morphogenesis, peptidoglycan synthesis arrest, and cell division inhibition in *S. suis*

**Fig 1:**
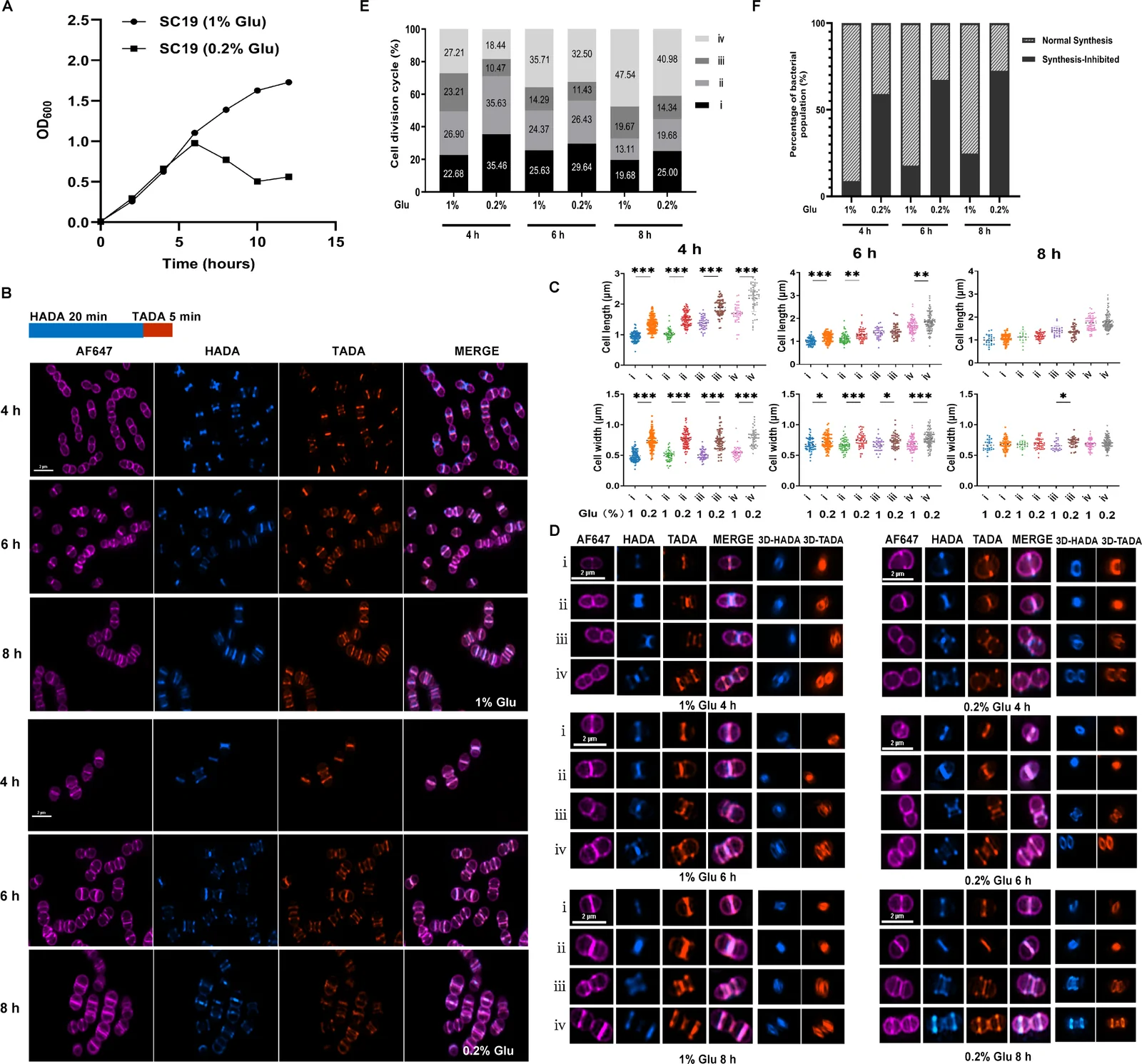
Glucose starvation alters cell morphology, arrests peptidoglycan synthesis, and disrupts cell division stage distribution in *S. suis*. (**A**) Growth curves of SC19 under normal and glucose-limited conditions. Growth curves of wild-type SC19 in complete CDM (1% glucose) vs glucose-starvation CDM (0.2% glucose), with bacterial growth monitored by OD₆₀₀ measurement at indicated time points and three independent biological replicates performed. (**B**) 3D-SIM imaging of SC19 cell morphology under different glucose conditions. Three-color 3D-SIM images of SC19 cells sequentially pulse-labeled with HADA (20 min) and TADA (5 min) to visualize nascent PG and counterstained with AF647 to delineate the total cell wall. Samples were taken at 4, 6, and 8 h under 1% or 0.2% glucose CDM. Scale bar, 2 µm. (**C**) Quantification of SC19 cell morphological parameters. Quantification of cell length and cell width from the SIM images in (**B**). Individual data points (*n* ≥ 300 cells per condition) are shown in jittered scatter plots, with median and interquartile range indicated. Statistical significance was determined by Student’s *t*-test. (**D**) 3D-SIM imaging of nascent PG synthesis in SC19 cells. Representative three-color merge images show the subcellular localization of HADA, TADA, and AF647 in SC19 cells cultured in CDM with 1% or 0.2% glucose, harvested at 4 h, 6 h, and 8 h. 3D-SIM reveals the localization pattern of newborn PG across four cell division stages grouped by PG remodeling patterns: (i) peripheral synthesis, (ii) septal synthesis, (iii) early constriction, and (iv) late constriction. Scale bar, 2 µm. (**E**) Quantitative distribution of cells across the four cell division stages. Quantification was performed at 4 h, 6 h, and 8 h in CDM with 1% or 0.2% glucose. Statistical significance of the difference in cell stage distribution between the glucose starvation group and normal glucose control group at each matched time point was determined by Pearson’s chi-square test. (**F**) Quantification of PG synthesis-arrested cell proportion. Quantification of the proportion of cells with active PG synthesis vs arrested PG synthesis at 4 h, 6 h, and 8 h in CDM with 1% or 0.2% glucose. Cells with arrested PG synthesis were defined by persistent colocalization of 3D-reconstructed HADA and TADA signals in PG synthesis regions. Statistical significance was determined by Pearson’s chi-square test between the glucose starvation group and normal glucose control group at each matched time point. *P < 0.05, **P < 0.01, ***P < 0.001.

To assess how carbon limitation affects growth, cell morphology, and PG biogenesis of *S. suis*, we cultivated wild-type SC19 strain in complete chemically defined medium (CDM) containing 1% glucose and in glucose-limited CDM containing 0.2% glucose, respectively. As shown in Fig. 1A, both cultures exhibited comparable growth for the first 6 h; thereafter, the OD₆₀₀ of the 0.2% glucose culture began to decline, while the 1% glucose culture continued to grow. To further characterize the morphological and PG synthesis changes induced by glucose limitation, cells were stained with Alexa Fluor 647 dye (AF647) to mark the cell outline and pulse-labeled with HCC-amino-D-alanine (HADA) (20 min) followed by TAMRA-amino-D-alanine (TADA) (5 min) to visualize newly synthesized PG. HADA and TADA are fluorescent D-amino acid (FDAA) probes that specifically incorporate into newly synthesized PG, allowing us to visualize the spatial and temporal patterns of septal and peripheral PG insertion. Time points of 4, 6, and 8 h were selected as determined by growth-curve analysis. 4 and 6 h correspond to the exponential growth phase when cell division is active, whereas 8 h marks the transition to slower growth and incipient nutrient limitation. Based on characteristic PG-remodeling patterns, cells were assigned to four stages: (*i*) peripheral synthesis, (*ii*) septal synthesis, (*iii*) early constriction, and (*iv*) late constriction. At both 4 h and 6 h, cells grown in 0.2% glucose-containing CDM exhibited significantly greater length and width across stages *i–iv* relative to the 1% glucose control, except at stage *iii* (6 h) where lengths were similar but widths remained larger in the 0.2% group (Fig. 1B and C). At 8 h, however, as nutrients became progressively limiting, the phenotypic differences between the 0.2% and 1% glucose groups were reduced. Lengths and widths measured across stages *i–iv* were more similar between conditions (Fig. 1B and C).

To examine in detail how PG architecture is impacted by glucose starvation, 3D SIM images of fluorescence-labeled cells were acquired, with representative images presented in Fig. 1D. Cells with arrested PG synthesis were defined as those exhibiting colocalization of 3D-reconstructed HADA (3D-HADA) and TADA (3D-TADA) signals within PG synthesis regions. The proportion of these synthesis-arrested cells was subsequently quantified and reported as a percentage of the total cell number at each time point (detailed classification criteria are described in Materials and Methods). Using this criterion, direct comparison of 3D SIM results at the 4 h time point revealed a marked difference between the 1% glucose group and the 0.2% glucose group. Nearly complete colocalization of 3D-HADA and 3D-TADA signals was detected in the 0.2% glucose group. Similar results were observed at the 6 h and 8 h time points. This finding suggested that the progression of PG synthesis was blocked without significant forward advancement. In contrast, a distinct spatial separation of 3D-HADA and 3D-TADA signals was observed in the 1% glucose control group, as shown in Fig. 1D. Compared with the 1% glucose control group, the 0.2% glucose group showed a significantly higher proportion of cells arrested at stages *i* and *ii* at the indicated time points. Specifically, the proportion of cells at stage i was markedly elevated at 4 h (Pearson’s chi-square test, *P* < 0.001), and the proportion of cells at stage *ii* was significantly increased at both 4 h and 8 h (Pearson’s chi-square test, *P* < 0.05 for both) in the 0.2% glucose group (Fig. 1E). Further analysis confirmed that the 0.2% glucose group exhibited a 3- to 7-fold higher proportion of synthesis-arrested cells than the 1% glucose control group at all matched time points, with highly significant differences detected at 4 h, 6 h, and 8 h (Pearson’s chi-square test, all *P* < 0.001) (Fig. 1F). Collectively, these observations suggest that glucose starvation modulates cellular geometry by inhibiting PG biogenesis, leading to cell division arrest and a spherical morphogenesis.

### Glucose starvation induces (p)ppGpp accumulation and RelA upregulation in *S. suis*

**Fig 2:**
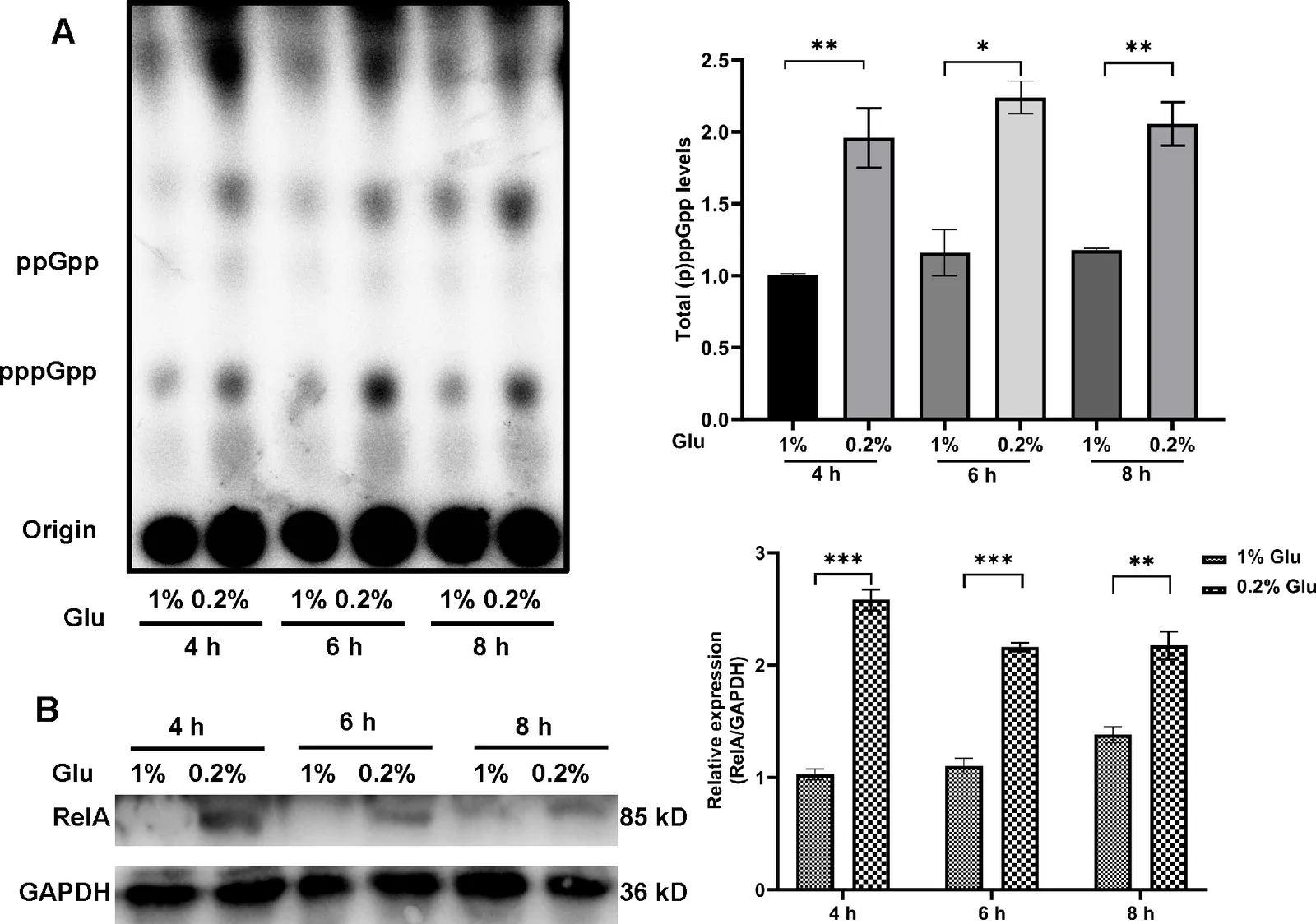
Glucose starvation induces RelA upregulation and (**P**)ppGpp accumulation in *S. suis.* (**A**) Quantification of intracellular (P)ppGpp levels in SC19. Quantification of intracellular (P)ppGpp levels in SC19 cells resuspended in MOPS-CDM supplemented with 1% glucose (normal glucose condition) or 0.2% glucose (glucose starvation condition), harvested at 4 h, 6 h, and 8 h. Intracellular (P)ppGpp was detected and quantified by [γ-^32^P]-ATP-based TLC. Statistical significance between the glucose starvation group and the normal glucose control group at each matched time point was determined using an unpaired Student’s *t*-test. (**B**) Western blot analysis of RelA protein abundance. Western blot analysis of RelA protein abundance in cells harvested at the indicated time points, with GAPDH used as the loading control. All experiments were performed with at least three independent biological replicates. Statistical differences were evaluated using an unpaired Student’s *t*-test. *P < 0.05, **P < 0.01, ***P < 0.001.

To test whether the starvation phenotypes correlate with (p)ppGpp alarmone signaling, intracellular (p)ppGpp was quantified by [γ-^32^P]-ATP-based thin-layer chromatography (TLC). It was shown in Fig. 2A that cells grown in 0.2% glucose MOPS-CDM accumulated substantially higher (p)ppGpp at 4, 6, and 8 h compared with those in the 1% glucose, with the (p)ppGpp levels in the 0.2% glucose group being extremely significantly higher than those in the 1% glucose group at 4 h and 8 h, and significantly higher at 6 h. A temporal association between stage distribution and RelA dynamics was noted. In 0.2% glucose, the proportion of cells transitioning from stage *i–ii* to stage *iii–iv* increased between 4 and 8 h, coinciding with a reduction in RelA protein abundance over the same interval (Fig. 1E; Fig. 2B). This pattern is consistent with a reversible, time-dependent adaptation in which an early RelA/(p)ppGpp elevation is associated with PG synthesis arrest, followed by partial recovery as RelA levels fall. These data demonstrate that glucose limitation drives RelA upregulation and concomitant (p)ppGpp accumulation in *S. suis*, suggesting a potential link between (p)ppGpp accumulation and the observed inhibition of PG synthesis and cell-division arrest.

### Elevated (p)ppGpp is sufficient to recapitulate carbon-starvation-associated morphological and PG synthesis defects independent of starvation in *S. suis*

**Fig 3:**
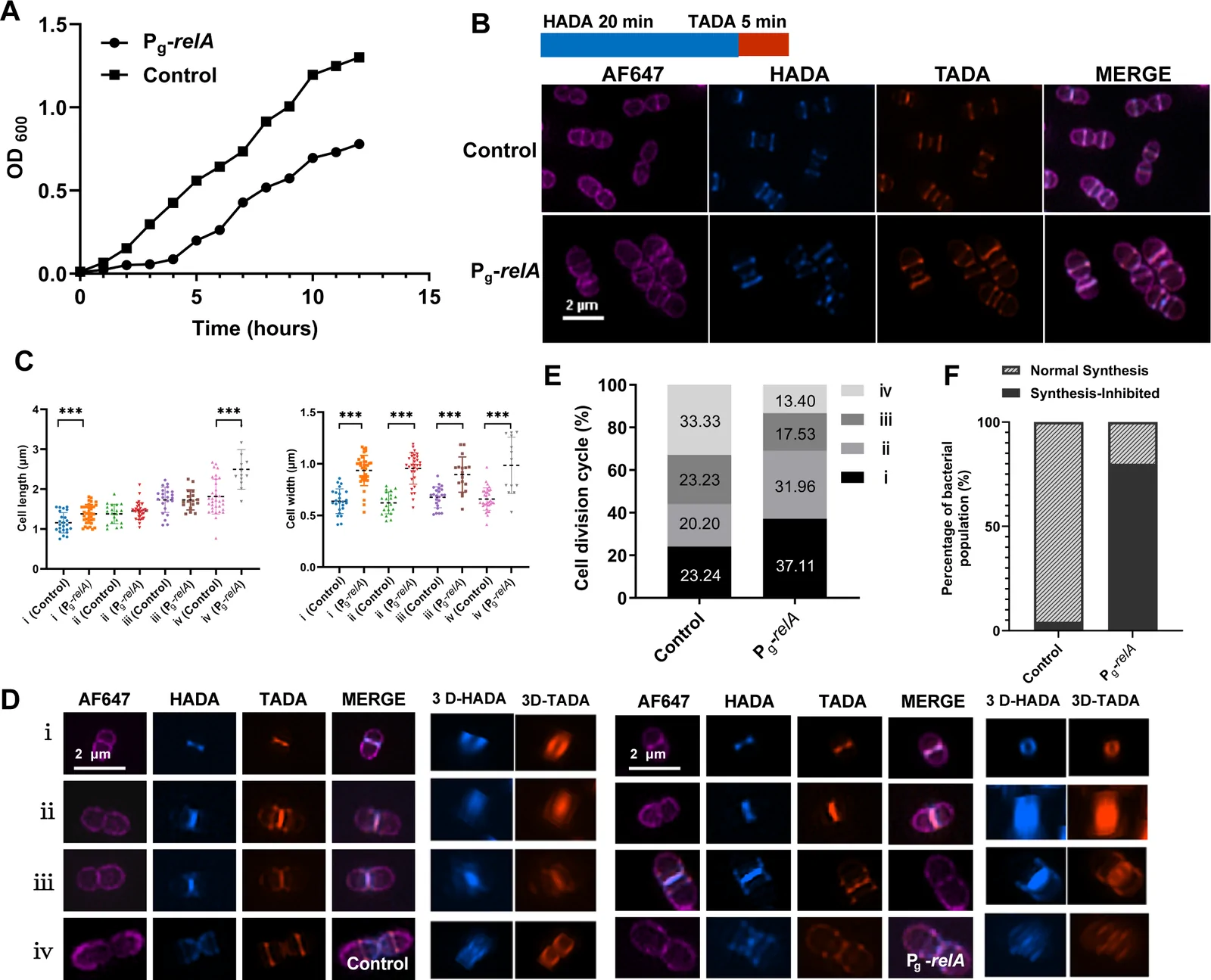
Constitutive RelA overexpression elevates (P)ppGpp and phenocopies carbon-starvation-associated PG defects. (**A**) Growth curves of control and RelA overexpression strains. Growth curves of the empty vector control strain and the constitutive RelA overexpression strain (P_g_-*relA*) cultured in complete CDM supplemented with 1% glucose. Bacterial growth was monitored by measuring the optical density at 600 nm (OD₆₀₀) at the indicated time points. (**B**) 3D-SIM imaging of cell morphology in control and P_g_-*relA* strains. Representative three-color 3D structured illumination microscopy (3D-SIM) images of the control strain and P_g_-*relA* strain. Cells were sequentially pulse-labeled with HADA (20 min) and TADA (5 min) for dynamic visualization of nascent PG and counterstained with AF647 to delineate the intact cell envelope. Scale bar, 2 µm. (**C**) Quantification of cell morphological parameters in control and P_g_-*relA* strains. Quantification of cell length and cell width based on the 3D-SIM images in panel (**B**). Individual data points (*n* ≥ 300 cells per condition) are shown in jittered scatter plots, with median and interquartile range indicated. Statistical significance between the P_g_-*relA* group and control group was determined by Student’s *t*-test. (**D**) 3D-SIM imaging of nascent PG synthesis in control and P_g_-*relA* strains. Representative three-color merge 3D-SIM images showing the localization of HADA, TADA, and AF647 in the control and P_g_-*relA* strains cultured in 1% glucose CDM. 3D-SIM reveals the vertically oriented localization pattern of newborn PG across four cell division stages (i to iv, grouped by PG remodeling patterns). Scale bar, 2 µm. (**E**) Quantitative distribution of cells across cell division stages. Quantitative distribution of cells across division stages i to iv in the control and P_g_-*relA* strains. Statistical significance of the difference in cell stage distribution between the two groups was determined by Pearson’s chi-square test. (**F**) Quantification of PG synthesis-arrested cell proportion. Quantification of the proportion of cells with active PG synthesis vs arrested PG synthesis in the control and P_g_-*relA* strains. Cells with arrested PG synthesis were defined by persistent colocalization of 3D-reconstructed HADA and TADA signals in PG synthesis regions. Statistical significance of the difference in the proportion of synthesis-arrested cells between the two groups was determined by Pearson’s chi-square test. *P < 0.05, **P < 0.01, ***P < 0.001.

To test whether elevated (p)ppGpp is sufficient to block PG synthesis, an SC19 derivative P_g_-*relA* strain was constructed in which *relA* is driven by a strong constitutive promoter P_g_. Western blotting analysis confirmed markedly higher RelA expression in P_g_-*relA* cells than in the empty-vector control (SFig. 1A), and TLC assay showed a corresponding increase in intracellular (p)ppGpp in the P_g_-*relA* strain (SFig. 1B). Consistent with the cells cultured in glucose-starved conditions, P_g_-*relA* exhibited a significantly slower growth rate in CDM with 1% glucose relative to control cells without RelA overexpression (Fig. 3A). Morphometric analysis revealed that P_g_-*relA* cells were appreciably wider, while cell length remained largely unchanged, resulting in a more spherical morphology (Fig. 3B and C). Super-resolution fluorescent imaging of sequential HADA/TADA-labeled cells revealed increased 3D-HADA and 3D-TADA colocalization in P_g_-*relA* cells compared with the control group, suggesting that RelA-driven (p)ppGpp accumulation affects the progression of PG synthesis (Fig. 3D). Furthermore, a significantly greater proportion of P_g_-*relA* cells were arrested at stages i and ii of cell division, corresponding to cells stalled at the peripheral-to-septal PG synthesis transition, with highly significant differences determined by Pearson’s chi-square test (*P* < 0.001 for stage i and *P* < 0.01 for stage ii) (Fig. 3E). The fraction of cells exhibiting inhibited PG synthesis was also extremely significantly elevated in the P_g_-*relA* strain by Pearson’s chi-square test with *P* < 0.001 (Fig. 3F). Together, these data demonstrate that RelA-mediated (p)ppGpp elevation is sufficient to reproduce the PG synthesis arrest observed under carbon starvation.

To further validate the causal link between (p)ppGpp accumulation and the observed phenotypes, while excluding interference from endogenous alarmone synthesis, we constructed an ATc-inducible *relA* expression strain in a Δ*relA*Δ*relQ* double-deletion background. Validation assays confirmed loss of *relA*/*relQ* expression and markedly reduced basal (p)ppGpp levels in the Δ*relA*Δ*relQ* mutant, with no significant morphological defects under normal growth conditions (Fig. S2A through C). Using this validated background, we performed dose- and time-resolved ATc induction assays, which confirmed that RelA-mediated (p)ppGpp accumulation drives a progressive spherical morphological transition and PG synthesis arrest in a dose- and time-dependent manner. Full details of these validation assays are provided in Fig. S3 to S5.

### High (p)ppGpp levels repress *pbp2x* and *fts*X expression

**Fig 4:**
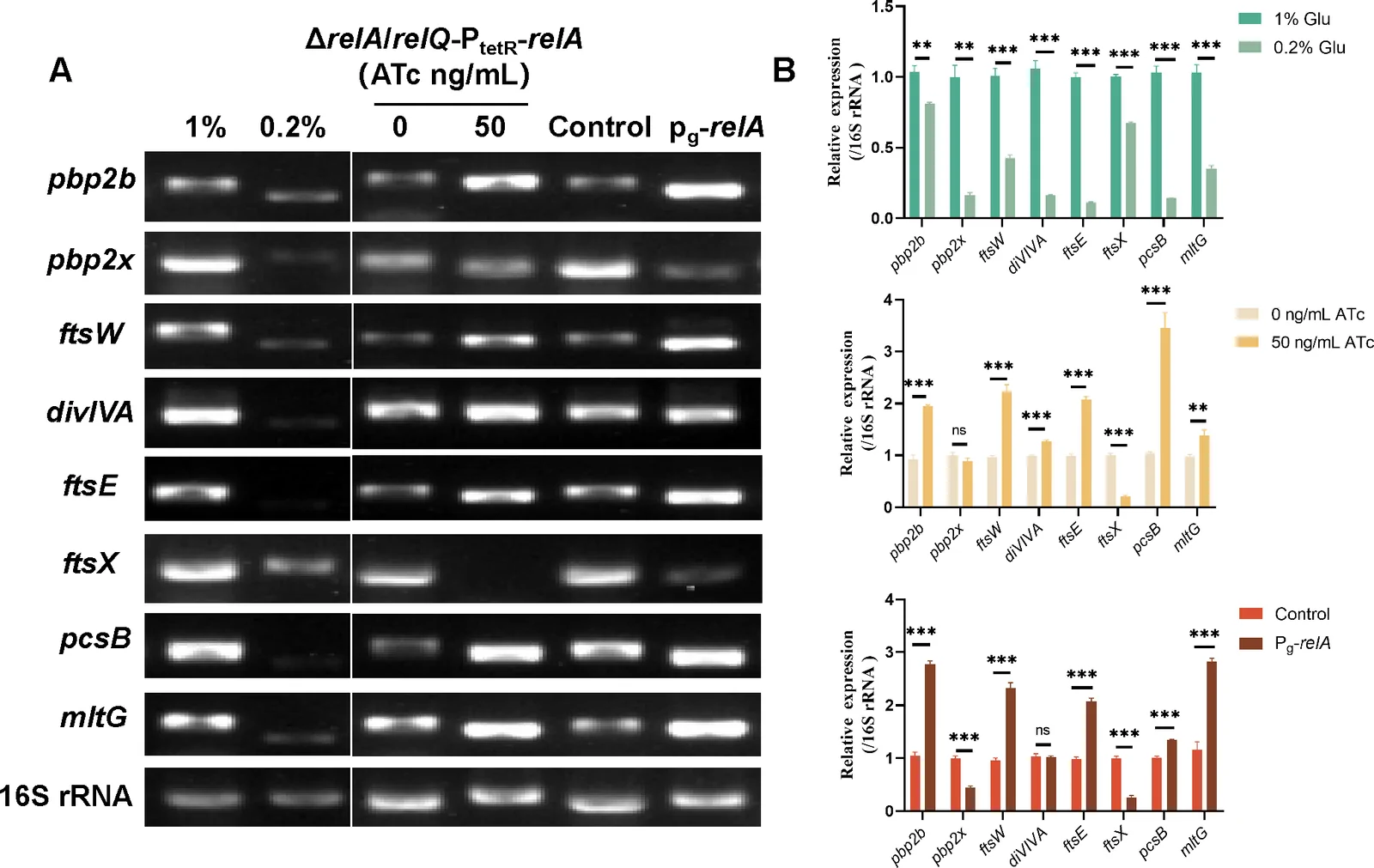
High (P)ppGpp levels downregulate transcription of cell division-related genes pbp2x and ftsX in *S. suis*. (**A**) Semi-quantitative PCR analysis of cell division-related gene transcription. Representative semi-quantitative PCR gel images showing amplicons of the cell division-related genes *pbp2x*, *ftsX, pbp2b*, *ftsW*, *divIVA*, *ftsE*, *pcsB,* and *mltG* under three matched experimental contrasts. These include normal glucose vs glucose starvation with 1% vs 0.2% glucose, uninduced vs ATc-induced RelA expression with 0 vs 50 ng/mL ATc in the Δ*relA*Δ*relQ* background carrying the ATc-inducible *relA* plasmid, and empty vector control vs constitutive RelA overexpression with control vs P_g_-*relA* strain. (**B**) Quantification of target gene transcription levels. Quantification of band intensities for *pbp2x*, *ftsX, pbp2b*, *ftsW*, *divIVA*, *ftsE*, *pcsB,* and *mltG* from the gels shown in (**A**). Statistical significance between the experimental group and corresponding control group for each contrast was determined by Student’s *t*-test. *P < 0.05, **P < 0.01, ***P < 0.001.

The expression of a panel of division-related genes (*pbp2x*, *ftsX*, *pbp2b*, *ftsW*, *divIVA*, *ftsE*, *pcsB,* and *mltG*) was analyzed using semi-quantitative PCR; the results showed that band intensities for *pbp2x* were visibly reduced in SC19 under low-glucose (0.2%) condition and in the P_g_-*relA* strain in normal growth conditions. *ftsX* was also downregulated under the low-glucose (0.2%) condition and in RelA overexpressing conditions, compared with the corresponding controls (Fig. 4A and B). In contrast, the remaining genes (*pbp2b*, *ftsW*, *divIVA*, *ftsE*, *pcsB,* and *mltG*), although expressed at lower levels under 0.2% glucose than under 1% glucose, did not display downregulation upon RelA/(p)ppGpp elevation (Fig. 4A and B). Given that RelA overexpression and (p)ppGpp accumulation phenocopied the glucose-starvation-associated spherical morphology and PG synthesis arrest, and that only *ftsX* shows consistent downregulation across all three models, while *pbp2x* was downregulated in two of the three models, these data suggest that *pbp2x* and *ftsX* could contribute to the (p)ppGpp-mediated inhibition of PG biogenesis.

### Deletion of *ftsX* or functional inhibition of PBP2x mimics the (p)ppGpp-dependent spherical phenotype in *S. suis*

**Fig 5:**
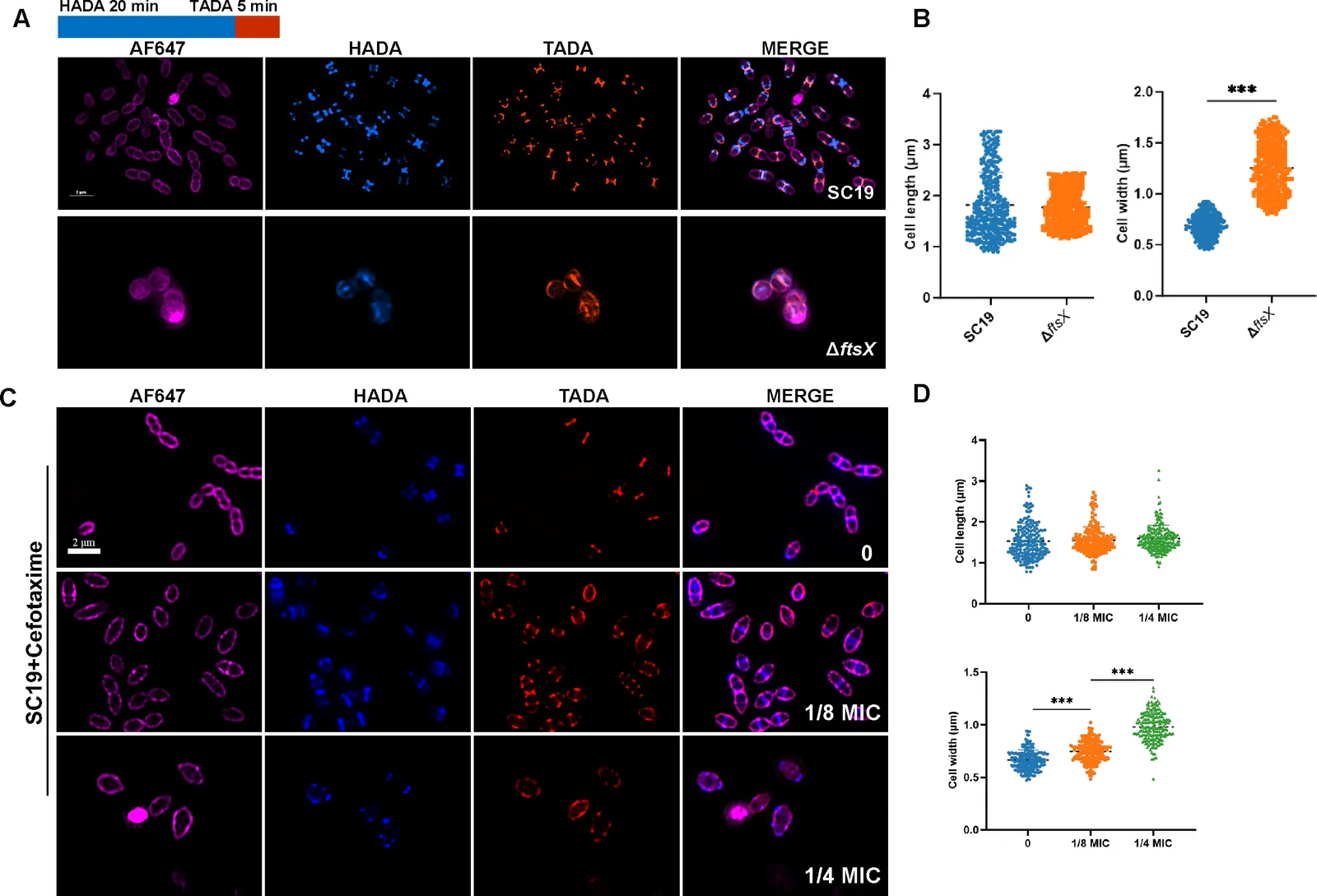
Deletion of *ftsX* or functional inhibition of PBP2x induces spherical morphogenesis in *S. suis*. (**A**) SIM imaging of wild-type SC19 and Δ*ftsX* mutant. Representative SIM images comparing wild-type SC19 and the isogenic Δ*ftsX* mutant. Cells were counterstained with AF647 to delineate the total cell wall. Scale bar, 2 µm. (**B**) Quantification of morphological parameters of wild-type SC19 and Δ*ftsX* mutant. Quantification of cell length and cell width from the SIM images in (**A**). Individual data points (*n* ≥ 300 cells per condition) are shown with median and interquartile range indicated in jittered scatter plots. Statistical significance between the Δ*ftsX* mutant and wild-type SC19 was determined by Student’s *t*-test. (**C**) SIM imaging of SC19 cells treated with sub-MIC cefotaxime. Representative SIM images of mid-log phase SC19 treated with vehicle (0), 1/8 × MIC (0.00, 390, 625 μg/mL) or 1/4 × MIC (0.0, 078, 125 μg/mL) cefotaxime for 2 h. The MIC of cefotaxime for SC19 is 0.03,125 μg/mL. Cells were sequentially pulse-labeled with HADA (20 min) and TADA (5 min) to visualize nascent PG and counterstained with AF647 to delineate the total cell wall. Scale bar, 2 µm. (**D**) Quantification of morphological parameters of cefotaxime-treated SC19 cells. Quantification of cell length and cell width from the SIM images in (**C**). Individual data points (*n* ≥ 300 cells per condition) are shown with median and interquartile range indicated in jittered scatter plots. Statistical significance among different cefotaxime concentration groups was determined by one-way ANOVA followed by Tukey’s multiple comparisons test. *P < 0.05, **P < 0.01, ***P < 0.001.

To test whether FtsX functions as a downstream effector in the signaling axis linking glucose limitation to (p)ppGpp accumulation and subsequent spherical morphogenesis, the morphology of an *ftsX* deletion mutant was examined. As shown in Fig. 5A, Δ*ftsX* cells exhibited a pronounced spherical morphology compared with the wild-type SC19 cells. Quantification of cell dimensions revealed that the Δ*ftsX* strain exhibited a significant increase in cell width while cell length remained largely unchanged (Fig. 5B). It was next investigated whether PBP2x plays a role in producing the spherical morphology. Because *pbp2x* is an essential gene for cell viability and a stable null mutant cannot be generated, we employed a pharmacological strategy using cefotaxime, a third-generation cephalosporin that preferentially targets PBP2x in *Streptococci*, to impair PBP2x activity (23). The mid-log phase SC19 culture was treated with 0, 1/8 × MIC, or 1/4 × MIC cefotaxime for 2 h, after which cell morphology was examined. Increasing sub-MIC cefotaxime concentrations produced a clear dose-dependent rounding of SC19 cells (representative images in Fig. 5C) and morphometric quantification confirmed increased cell width, while cell length remained largely unchanged (Fig. 5D). Therefore, deletion of *ftsX* and partial impairment of PBP2x function both resulted in a spherical morphology, suggesting that these PG biogenesis components could contribute to the (p)ppGpp-associated rounding phenotype.

### Glucose limitation induces conserved morphological changes and downregulation of peptidoglycan synthesis-related genes across bacterial species

**Fig 6:**
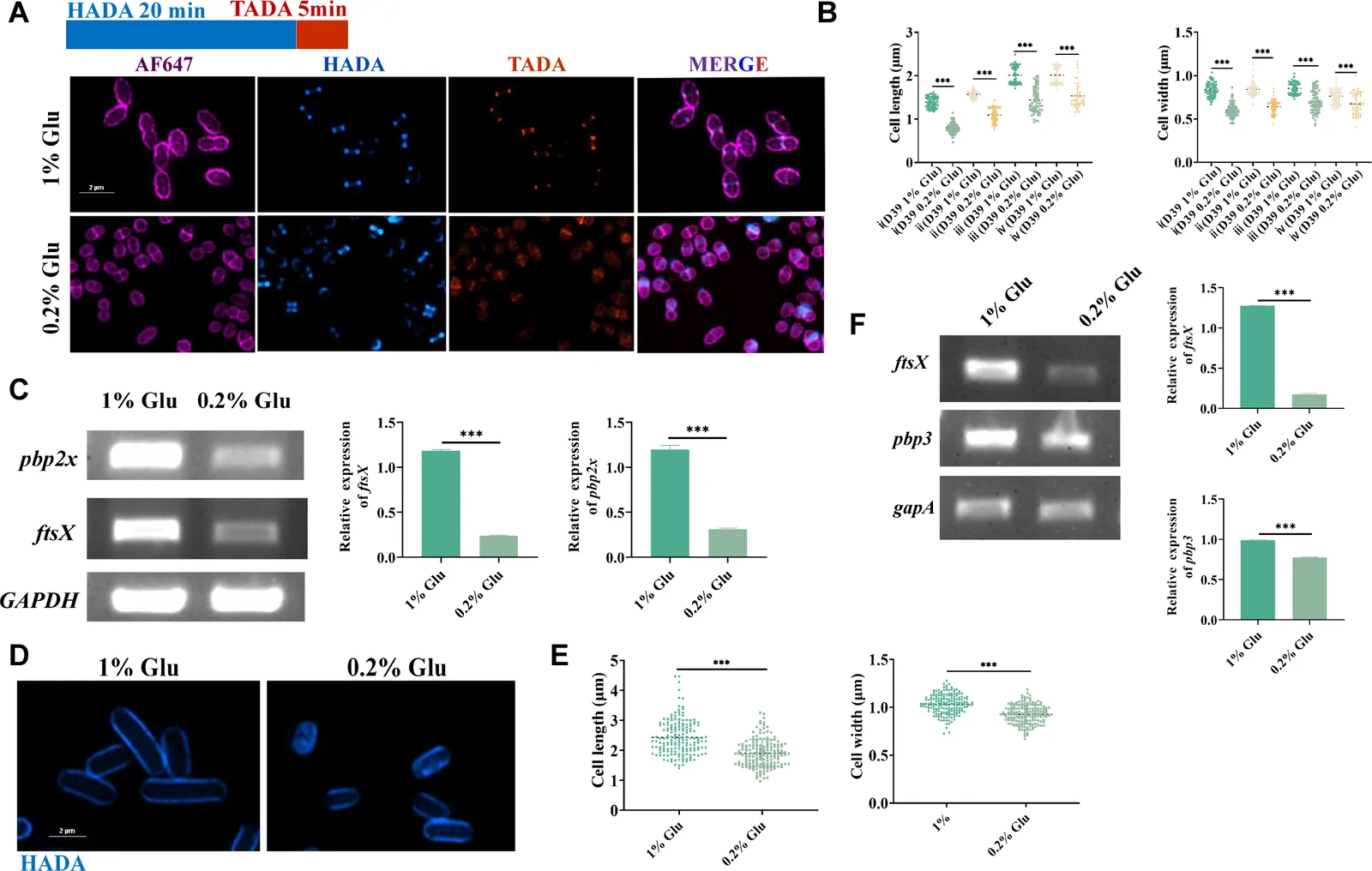
Glucose limitation induces conserved morphological changes and downregulation of peptidoglycan (PG) synthesis-related genes in *Streptococcus pneumoniae* and *Escherichia coli*. (**A**) SIM imaging of *S. pneumoniae* D39 cell morphology under different glucose conditions. Representative three-color SIM images of *S. pneumoniae* D39 cells. Cells were sequentially pulse-labeled with HADA (20 min) and TADA (5 min) to visualize nascent PG and counterstained with AF647 to delineate the total cell wall. Samples were harvested after growth in CDM containing 1% or 0.2% glucose. Scale bar, 2 µm. (**B**) Quantification of D39 cell morphological parameters. Quantification of cell length and cell width from the SIM images in (**A**). Individual data points (*n* ≥ 300 cells per condition) are shown with median and interquartile range indicated in jittered scatter plots. Statistical significance between the 0.2% glucose group and 1% glucose control group was determined by Student’s *t*-test. (**C**) Semi-quantitative PCR analysis of *pbp2x* and *ftsX* transcription in *S. pneumoniae*. Semi-quantitative PCR and corresponding quantification showing reduced *pbp2x* and *ftsX* transcript signals in the 0.2% glucose condition relative to the 1% glucose control condition. Statistical significance between the two groups was determined by Student’s *t*-test. (**D**) SIM imaging of *E. coli* W3110 cell morphology under different glucose conditions. Representative SIM images of *E. coli* W3110 cells labeled with HADA. Cells were grown in CDM containing 1% or 0.2% glucose. Scale bar, 2 µm. (**E**) Quantification of W3110 cell morphological parameters. Quantification of cell length and cell width from the SIM images in panel (**D**). Individual data points (*n* ≥ 300 cells per condition) are shown with median and interquartile range indicated in jittered scatter plots. Statistical significance between the 0.2% glucose group and 1% glucose control group was determined by Student’s *t*-test. (**F**) Semi-quantitative transcriptional analysis of *pbp3* and *ftsX* in *E. coli*. The assay shows reduced transcript abundance of target genes in the 0.2% glucose condition relative to the 1% glucose control condition. Statistical significance between the two groups was determined by Student’s *t*-test. *P < 0.05, **P < 0.01, ***P < 0.001.

To determine whether the glucose-limitation phenotype observed in *S. suis* extends to other bacterial species, we first analyzed morphological and transcriptional changes in the Gram-positive *Streptococcus pneumoniae* D39 under the same CDM conditions used above. SIM analysis after AF647 dye staining and sequential HADA/TADA labeling revealed that low glucose availability drove *S. pneumoniae* cells toward a more rounded morphology. Specifically, cells grown in 0.2% glucose-containing CDM exhibited an overall increase in sphericity compared with those in CDM containing 1% glucose (Fig. 6A and B). Semi-quantitative transcriptional analysis revealed that *pbp2x* and *ftsX* transcript signals were significantly reduced in the 0.2% glucose condition compared with those in normal condition (1% glucose) (Fig. 6C).

We next examined whether a related (p)ppGpp-dependent morphological response occurs in the Gram-negative model organism *Escherichia coli* W3110 under matched CDM glucose conditions. Consistent with prior reports that rod-shaped bacteria tend to become shorter under nutrient limitation (24, 25), SIM imaging of HADA-labeled *E. coli* cells revealed significant morphological changes under glucose limitation (0.2% vs 1% glucose) (Fig. 6D and E). A Δ*relA*Δ*spoT* double mutant was constructed in the *E. coli* W3110 background to enable controlled reconstitution of (p)ppGpp synthesis, with the validation of the mutant strain provided in SFig. 6A–C. Dose- and time-resolved RelA induction assays were further performed in the Δ*relA*Δ*spoT* background to validate the causal effect of (p)ppGpp on cell morphological changes, with related results provided in SFig. 7. Finally, transcriptional assays revealed that *pbp3* (the *S. suis pbp2x* homolog) and *ftsX* transcript levels were significantly reduced under 0.2% glucose compared with the 1% glucose control group (Fig. 6F). The regulatory effect of (p)ppGpp on these genes was further validated by IPTG induction assays, with results provided in SFig. 7G. Taken together, these observations demonstrate that glucose limitation drives conserved morphological changes and downregulation of the key PG synthesis-related genes *pbp2x*/*pbp3* and *ftsX* across both Gram-positive and Gram-negative bacterial species, supporting the universality of the (p)ppGpp-dependent morphological regulatory axis identified in *S. suis*.

## DISCUSSION

Here, we report that in the zoonotic pathogen *Streptococcus suis*, a RelA-dependent rise in intracellular (p)ppGpp is triggered by glucose limitation, and this elevation appears tightly coupled to slowed growth, cell division arrest, and a highly reproducible spherical morphotype. SIM imaging showed this morphological transition stems from impaired progression of nascent PG synthesis, a phenotype fully recapitulated by two independent, orthogonal genetic manipulations to elevate intracellular RelA/(p)ppGpp levels. Targeted deletion of *ftsX* and sub-inhibitory cefotaxime treatment to impair PBP2x function both phenocopied the rounded cell morphology, suggesting these two core PG synthesis factors could contribute to the (p)ppGpp-dependent shape change. Transcriptional profiling across three independent experimental models (glucose starvation, ATc-induced RelA expression, and constitutive P_g_*-relA* overexpression) suggests that *pbp2x* and *ftsX* appear to be the only cell division-related genes consistently downregulated by elevated (p)ppGpp, with conserved morphological and transcriptional signatures observed in *Streptococcus pneumoniae* and *Escherichia coli*. Together, our data suggest glucose starvation drives PG arrest and spherical morphogenesis via RelA-mediated (p)ppGpp accumulation, which acts, at least in part, through the transcriptional downregulation of *pbp2x* and *ftsX*.

Environmental and nutrient stresses are well established to drive rapid, adaptive remodeling of bacterial envelope architecture and PG metabolism (26). Starvation is a well-documented driver of envelope plasticity, and phylogenetically diverse bacteria commonly adopt a compact coccoid shape, suspend active cell division, and upregulate stress tolerance pathways to survive nutrient scarcity (3, 27). Prior work in the field frames this pausing of the cell division program as a core adaptive trade-off, whereby cells downregulate the energetically costly processes of septation and PG precursor synthesis to conserve limited nutrient resources and reduce the risk of catastrophic fatal division errors under stress (28). Consistent with this framework, our study observed glucose limitation induced an increase in cell width, sphericity, and impaired PG synthesis in *S. suis*, a phenotype we interpret as a regulated adaptive division pause rather than non-specific cytotoxicity, with this conserved response detected across three distinct bacterial species.

Our work extends the current understanding of (p)ppGpp function by defining a potential direct regulatory link between (p)ppGpp accumulation and the inhibition of PG synthesis, rather than a global non-specific suppression of cell wall synthesis. The (p)ppGpp-driven stringent response is a highly conserved bacterial program that reallocates cellular resources away from growth and proliferation and toward stress survival by reshaping genome-wide transcription and central metabolism. Recent work has emphasized its central role in coordinating bacterial adaptation to both carbon and amino acid downshifts (5). Our two independent genetic strategies for controlled (p)ppGpp elevation in *S. suis*, alongside a complementary IPTG-inducible RelA expression model in *E. coli*, strengthen the causal link between RelA-mediated (p)ppGpp accumulation and cell division arrest. On this basis, it is proposed that (p)ppGpp functions as a programmable metabolic brake that reallocates cellular resources away from PG synthesis and proliferation under carbon stress.

Mechanistically, (p)ppGpp can influence PG synthesis at multiple regulatory tiers ranging from transcriptional reprogramming of PG biosynthetic and cell division genes, to metabolic rebalancing of PG precursor pools, and post-transcriptional or post-translational regulation of key synthetic enzymes. This provides a robust mechanistic basis for the observed reductions in *pbp2x* and *ftsX* transcript abundance under (p)ppGpp-elevating conditions. This link is further supported by experimental validation performed in this study. The progressive rounding phenotype observed in glucose-starved cells was replicated via selective pharmacological inhibition of PBP2x with sub-MIC cefotaxime, a finding that suggests PBP2x deficiency is a likely key contributor to this morphological transition in *S. suis*. This spherical morphotype is consistent with observations in other ovococcal bacteria, including the conserved role of the FtsEX complex in streptococcal septal PG remodeling (12). The striking divergent phenotype of PBP2x inactivation between *S. suis* and *S. pneumoniae* (29) likely reflects species-specific differences in the functional coupling between the septal and peripheral PG synthesis machineries. It is emphasized that while phenotypic sufficiency of *pbp2x*/*ftsX* impairment for the (p)ppGpp-dependent morphology is supported by the present data, it is not formally proven that these genes are necessary downstream mediators of the alarmone effect. Definitive genetic epistasis analysis will be required to formally establish direct causality.

In conclusion, our study elucidates a potential RelA-dependent signaling axis that bridges nutritional status and cell wall morphogenesis in *S. suis*, a link which remains poorly defined in the existing literature. Through the integration of glucose starvation assays, controlled genetic titration of intracellular (p)ppGpp levels, and functional validation of PBP2x and FtsX, convergent evidence suggests that (p)ppGpp accumulation acts as a metabolic brake on PG synthesis progression under carbon stress. The conservation of this morphological and transcriptional signature across diverse Gram-positive and Gram-negative bacterial taxa underscores the fundamental nature of this metabolic-structural coupling. This work identifies a potential molecular link between metabolic nutrient sensing and the core bacterial cell division machinery, providing a robust framework for future investigations into the hierarchical regulation of bacterial growth, and offering promising new targets to disrupt bacterial stress adaptation and persistence during host infection.

## MATERIALS AND METHODS

### Bacterial strains and culture conditions

SC19 and its genetically engineered derivatives were cultivated in tryptic soy broth (TSB; Difco) or on tryptic soy agar (TSA; Difco) supplemented with 10% (vol/vol) fetal bovine serum (Every Green, China) at 37°C for routine propagation, or at 28°C for temperature-sensitive recombination procedures. The *S. pneumoniae* strains are derived from the capsule-deficient mutant of the D39 strain (*S. pneumoniae* D39 Δ*cps rpsL*^+^). Strains from *S. pneumoniae* were routinely grown in brain heart infusion (BHI; Difco) medium at 37°C in 5% CO_2_ without shaking. A chemically defined medium (CDM) was prepared according to Vanderijn and Kessler (30) and modified as required. For standard growth assays, cultures were maintained in CDM containing 1% (wt/vol) glucose (normal-glucose condition), whereas glucose-limitation experiments were performed in CDM supplemented with 0.2% (wt/vol) glucose. For *in vivo* ^32^P-labeling, a phosphate-free MOPS-CDM formulation was employed, consisting of CDM devoid of phosphate salts but supplemented with 40 mM MOPS, 4 mM Tricine, 0.28 mM K₂SO₄, and 50 mM NaCl.

*Escherichia coli* DH5α was used for routine cloning and BL21(DE3) for heterologous protein expression; for general handling, these *E. coli* strains were propagated in Luria-Bertani (LB) broth or on LB agar and antibiotics were added as appropriate (spectinomycin 100 µg/mL, kanamycin 50 µg/mL, chloramphenicol 100 µg/mL, ampicillin 150 µg/mL). For glucose-starvation validation experiments, *E. coli* W3110 and *Streptococcus pneumoniae* D39 were cultured in the same CDM used for *S. suis*, supplemented with either 1% (wt/vol) or 0.2% (wt/vol) glucose as specified. *S. pneumoniae* D39 cultures in CDM were incubated at 37°C with 5% CO₂ for liquid assays. Bacterial growth was monitored at 600 nm (OD₆₀₀) using an Eppendorf BioSpectrometer basic (Eppendorf AG, Germany). A complete list of bacterial strains and plasmids used in this study is provided in Tables S1 and S2.

### Plasmid construction and mutant generation

Oligonucleotide primers used for plasmid assembly and strain construction are listed in Table S3. Unless otherwise specified, all recombinant plasmids were generated via single-step homologous recombination using the ClonExpress MultiS kit (Vazyme, Nanjing, China) following the manufacturer’s protocol (31). The pSET2-P_g_-RelA and anhydrotetracycline (ATc)-inducible RelA expression plasmids were constructed according to methodologies previously established in our laboratory (32). pET28a was used for protein expression. The temperature-sensitive suicide vector pSET4s was employed to generate the SC19 double-deletion mutant (Δ*relA*Δ*relQ*). The *ftsX* deletion mutant (Δ*ftsX*) in *S. suis* was obtained from a previously constructed and stored in our laboratory.

For *E. coli*, *relA* and *spoT* were sequentially deleted in W3110 using λ-Red recombineering. Antibiotic resistance cassettes (flanked by FRT sites) were amplified with homology extensions and introduced via pKD46-mediated recombination; resistance markers were removed by FLP recombinase (pCP20) to generate the unmarked Δ*relA*Δ*spoT* double mutant, which was confirmed by diagnostic PCR and Sanger sequencing. An IPTG-inducible *relA* expression plasmid was constructed by cloning the *E. coli relA* open reading frame into an IPTG-responsive vector (pQE80) and introducing the plasmid into the Δ*relA*Δ*spoT* background; induction conditions used in phenotypic assays are specified in the relevant Materials and Methods subsections (IPTG 0–1 mM, dose/time ranges as indicated in figure legends). All constructs and mutants were validated by colony PCR and Sanger sequencing, and where appropriate by phenotype (growth, alarmone accumulation and/or microscopy).

### Western blotting

*S. suis* cell pellets were washed twice with ice-cold PBS and resuspended in lysis buffer containing 0.1 M Tris-HCl (pH 7.5), 150 mM NaCl, 1% Triton X-100, 0.2 mg/mL protease inhibitor cocktail, and 500 U/mL mutanolysin (Sigma-Aldrich). Cells were lysed by sonication on ice, and cell debris was removed by centrifugation. Protein concentrations were quantified and equalized across samples. Equal amounts of total protein were mixed with SDS loading buffer, boiled, and separated on 12% SDS-PAGE gels. Proteins were transferred onto PVDF membranes (Millipore) by wet transfer using a standard tank blotting system (Bio-Rad). Membranes were blocked overnight at 4°C in PBS-0.05% Tween-20 containing 5% non-fat milk, followed by incubation with mouse-derived anti-RelA polyclonal antibody (1:1,000, in-house), generated against purified recombinant RelA. After washing, membranes were incubated with HRP-conjugated goat anti-mouse IgG (Proteintech, 1:5,000). Signals were developed using ECL substrate and visualized on a digital chemiluminescence imaging system (Bio-Rad).

### Fluorescence microscopy

For visualization of nascent PG and cell morphology, structured‐illumination microscopy (SIM) was performed on a Nikon SIM system (Nikon Instruments, Japan). For dynamic labeling, *S. suis* and *S. pneumoniae* cells were first pulsed with HADA for 20 min, washed three times with PBS, and then pulsed with TADA for 5 min, each at 250 µM. *E. coli* samples were only labeled with HADA (20 min). To delineate the entire cell envelope (*S. suis* and *S. pneumoniae* cells), samples were incubated with 20 µg mL⁻¹ Alexa Fluor 647 NHS‐ester (Thermo Fisher) for 45 min at 37°C without shaking, followed by PBS washes. For *E. coli*, cells were only incubated with HADA for 20 min at 37°C, washed three times with PBS, and processed as below. Stained cells were mounted on 0.7% agarose pads and imaged under SIM. Channels were acquired using laser lines at 405 nm (HADA; emission 435–545 nm), 561 nm (TADA; emission 570–640 nm), and 647 nm (AF647; emission 663–738 nm). Acquisition settings (laser power, exposure, and z-step) were kept constant across comparative conditions unless otherwise stated in the figure legends.

### Image acquisition and image analysis

All images were acquired with identical microscope settings (laser power, exposure, and z-step) for comparable samples and reconstructed with the same parameters; quantitative analysis was performed on reconstructed 3D-SIM stacks using Fiji/ImageJ (v1.53). Cell outlines were segmented from the AF647 channel by automatic thresholding (Otsu) followed by watershed separation for touching cells, and objects outside the expected size range were excluded. For each segmented cell a PG region of interest (ROI) was defined as a band of ~300 nm centered on the geometric midline perpendicular to the long axis. 3D-HADA and 3D-TADA channels were background-subtracted (rolling ball radius = 50 px) and binarized using an intensity threshold derived from control images and then applied uniformly across conditions. Within the PG ROI, a colocalization fraction was calculated. This fraction is defined as the ratio of the number of pixels positive for both 3D-HADA and 3D-TADA to the number of pixels positive for either 3D-HADA or 3D-TADA. Based on this, metric cells were classified as PG synthesis-arrested when the PG colocalization fraction was ≥0.50, normal (active PG synthesis) when the PG colocalization fraction was ≤0.30, and intermediate/ambiguous when the colocalization fraction was between 0.30 and 0.50; intermediate cells were annotated but not included in the binary proportion statistics. Exact *n* values and statistical tests are reported in the figure legends.

### RNA extraction and reverse‐transcription PCR

Total RNA was extracted from samples using the SV Total RNA Isolation System (Promega). First-strand cDNA was synthesized with HiScript Q Select RT SuperMix (Vazyme). Cell division-related transcripts were amplified by endpoint PCR using gene-specific primers (Table S3) and 2 × Magic Green Taq SuperMix (Tolobio) in 20 μL reactions. Thermocycling conditions were 95°C for 10 min; 25–30 cycles of 95°C for 30 s, 55–60°C for 30 s, and 72°C for 1 min; with a final extension at 72°C for 10 min. PCR products were resolved on 2% agarose gels and visualized by ethidium bromide staining.

### (p)ppGpp quantification

Intracellular (p)ppGpp levels were determined by radiolabel–TLC as previously described (20), with modifications for *S. suis* and *E. coli*. Overnight cultures of *S. suis* or *E. coli* were diluted into fresh TSB (for *S. suis*) or LB (for *E. coli*) and grown to an OD₆₀₀ of 0.4–0.5. Cells were then washed and resuspended in MOPS-CDM medium supplemented with either 0.2% or 1% glucose, and the OD₆₀₀ was adjusted to 0.2. A 150 μL aliquot of this suspension was transferred to a fresh microcentrifuge tube and mixed with an equal volume of 150 μCi/mL H_3_[^32^P]O_4_. The mixture was incubated at 37 °C, and samples were collected at defined time points and immediately mixed with an equal volume of 13 M formic acid. After two cycles of rapid freeze-thaw in liquid nitrogen, 5 μL of the clarified lysate was spotted onto polyethyleneimine (PEI)-cellulose TLC plates and developed in 1.5 M KH₂PO₄ (pH 3.4). Plates were air-dried and exposed to a phosphor screen, and radiolabeled nucleotides were visualized and quantified using a phosphorimager (Fuji).

### Antibiotic susceptibility testing and cefotaxime treatment

The minimum inhibitory concentration (MIC) of cefotaxime for SC19 was determined by broth microdilution according to Clinical and Laboratory Standards Institute (CLSI) guidelines. Briefly, twofold serial dilutions of cefotaxime were prepared in sterile broth and inoculated with ~5 × 10^5^ CFU/mL of mid-log phase bacteria; plates were incubated under appropriate growth conditions, and the MIC was recorded as the lowest concentration showing no visible growth. Cefotaxime stock solutions were freshly prepared in sterile water immediately prior to use. For morphological perturbation assays, overnight cultures of SC19 were diluted into CDM and grown to mid-log phase (OD_600_ ≈ 0.2–0.4). Cultures were then adjusted to identical optical densities and exposed to cefotaxime at 0 (vehicle control), 1/8 × MIC, and 1/4 × MIC for 2 h at 37°C. Following treatment, aliquots were collected for SIM using the HADA/TADA sequential labeling protocol described in section Fluorescence microscopy. For microscopy samples, cells were washed twice with PBS, mounted on 0.7% agarose pads, and imaged under identical acquisition settings to permit quantitative morphometry. All cefotaxime treatments were performed in biological triplicate.

### Statistics and reproducibility

All experiments were performed with at least three independent biological replicates. For the quantitative analysis of cells, more than 300 single cells were randomly selected and analyzed for each experimental condition. These cells were isolated from three independent biological replicates, and cell aggregates were excluded during screening to ensure the objectivity and reliability of the quantitative data. Statistical analyses were conducted using GraphPad Prism 8 (GraphPad Software, USA). Depending on the experimental design, either Student’s *t*-test or one-way ANOVA followed by Tukey’s multiple comparisons test was used. A *P* value < 0.05 was considered statistically significant. Significance levels are denoted as follows: *P* < 0.05 (*), *P* < 0.01 (**), and *P* < 0.001 (***).

## SUPPLEMENTAL MATERIAL

Supplemental figures jb.00030-26-s0001.docx Fig. S1 to S7.

Tables S1 to S3 jb.00030-26-s0002.docx Table 1: Bacterial strains used in this study. Table 2: Plasmids used in this study. Table 3: Oligonucleotide primers used in this study.

ASM does not own the copyrights to Supplemental Material that may be linked to, or accessed through, an article. The authors have granted ASM a non-exclusive, world-wide license to publish the Supplemental Material files. Please contact the corresponding author directly for reuse.
