# Supplementary material for: RelA-mediated (p)ppGpp accumulation links glucose starvation to peptidoglycan synthesis arrest and spherical morphogenesis via PBP2x and FtsX in Streptococcus suis
Source: J Bacteriol. 2026 Jul 14;208(8):e00030-26. doi: 10.1128/jb.00030-26 (PMC13492137; doi:10.1128/jb.00030-26)
Supplement: Supplementary file 1 [file jb.00030-26-s0001.docx]

****Supplementary Figure 1. Constitutive RelA overexpression via the P_g_-*relA* construct elevates RelA protein levels and intracellular (p)ppGpp abundance in *Streptococcus suis.*****

****A.** Representative Western blot analysis of RelA protein expression in control and P_g_-*relA* overexpression strains, with GAPDH as the loading control. **B.** Representative TLC analysis of intracellular (p)ppGpp levels in control and P_g_-*relA* strains. Data are presented as mean ± SEM from three independent biological replicates.** **p* < 0.05, ***p* < 0.01, ****p* < 0.001.

****Supplementary Figure 2. Validation of the Δ*relA*Δ*relQ* double-deletion strain in** S. suis **SC19.****
**A.** Semi-quantitative PCR illustrating absence of detectable relA and relQ amplicons in the ΔrelAΔrelQ mutant. The parental SC19 strain shows robust bands for both targets. **B.** Representative radiolabeled TLC of intracellular nucleotides showing markedly lower (p)ppGpp accumulation in the ΔrelAΔrelQ mutant relative to SC19. **C.** Representative three-color SIM images of uninduced ΔrelAΔrelQ cells labeled with HADA (20 min), TADA (5 min) and counterstained with AF647, showed no significant differences in cell length and width compared with SC19 control. However, the mutant exhibited noticeably longer bacterial chains. **p* < 0.05, ***p* < 0.01, ****p* < 0.001.

****Supplementary Figure 3. ATc-inducible RelA expression in a Δ*relA*Δ*relQ* background drives dose- and time-dependent RelA accumulation and (p)ppGpp synthesis in *Streptococcus suis* SC19.****

****A.** Western blot of RelA in Δ*relA*Δ*relQ* strain carrying the ATc-inducible *relA* construct after 1 h induction with increasing ATc (0-70 ng/mL). GAPDH shown as loading control. **B.** Western blot of RelA in Δ*relA*Δ*relQ* strain carrying the ATc-inducible *relA* construct after addition of 50 ng/mL ATc (0-2 h). GAPDH shown as loading control. **C.** (p)ppGpp levels in Δ*relA*Δ*relQ* strain carrying the ATc-inducible *relA* construct after 1 h induction with increasing ATc (0-70 ng/mL). **D.** (p)ppGpp levels in Δ*relA*Δ*relQ* strain carrying the ATc-inducible *relA* construct after addition of 50 ng/mL ATc (0-2 h).** **p* < 0.05, ***p* < 0.01, ****p* < 0.001.

**Supplementary Figure 4. RelA induction phenocopies starvation-associated rounding in Δ*relA*Δ*relQ* cells.**

**A**. 3D-SIM imaging of cell morphology under gradient ATc induction. Representative three-color merge images acquired by 3D-SIM, showing the localization of HADA, TADA and AF647 in the Δ*relA*Δ*relQ* double mutant strain carrying an ATc-inducible *relA* expression plasmid. Cells were treated with gradient concentrations of ATc (0, 30, 50, 70 ng/mL) for 1 h, sequentially pulse-labeled with HADA (20 min) and TADA (5 min) for visualization of nascent PG, and counterstained with AF647 to delineate the intact cell envelope. Scale bar, 2 µm. **B**. 3D-SIM imaging of cell morphology across different RelA induction times. Representative three-color merge 3D-SIM images of the Δ*relA*Δ*relQ* inducible *relA* expression strain treated with 50 ng/mL ATc for 0 h, 1 h, 1.5 h and 2 h. Cells were labeled with HADA, TADA and AF647 using the same protocol described in panel (A). Scale bar, 2 µm. **C.** Quantification of spherical cell proportion under gradient ATc induction. Quantification of the percentage of spherical cells calculated from the 3D-SIM images in panel (A). Statistical significance between different ATc concentration groups was determined by one-way ANOVA followed by Tukey’s multiple comparisons test. **D**. Quantification of spherical cell proportion across different induction times. Quantification of the percentage of spherical cells calculated from the 3D-SIM images in panel (B). Statistical significance between different induction time groups was determined by one-way ANOVA followed by Tukey’s multiple comparisons test. **p* < 0.05, ***p* < 0.01, ****p* < 0.001.

**Supplementary Figure 5. ATc-induced RelA expression impairs progression of nascent PG synthesis.**

**A**. 3D-SIM imaging of nascent PG synthesis with or without ATc induction. Representative 3D-SIM images of the Δ*relA*Δ*relQ* double mutant strain carrying an ATc-inducible *relA* expression plasmid, treated with 0 ng/mL (uninduced control) or 50 ng/mL ATc for 1 h. Cells were sequentially pulse-labeled with HADA (20 min) and TADA (5 min) for visualization of nascent PG, and counterstained with AF647 to delineate the intact cell envelope. Rotated 3D reconstructions of the PG signal are shown for representative cells. Scale bar, 2 µm. **B**. 3D-SIM imaging of nascent PG synthesis across different induction times. Representative 3D-SIM images of the Δ*relA*Δ*relQ* inducible *relA* expression strain before (0 h) and 1 h after addition of 50 ng/mL ATc. Cells were labeled with HADA, TADA and AF647 using the same protocol described in panel (A). Scale bar, 2 µm. **C.** Quantification of PG synthesis-arrested cell proportion with or without ATc induction. Quantification of the proportion of cells with active PG synthesis versus arrested PG synthesis in the uninduced control group and 50 ng/mL ATc-induced group (1 h treatment) corresponding to panel (A). Cells with arrested PG synthesis were defined by persistent co-localization of 3D-reconstructed HADA and TADA signals in PG synthesis regions. Statistical significance of the difference in the proportion of synthesis-arrested cells between the two groups was determined by Pearson’s chi-square test. **D**. Quantification of PG synthesis-arrested cell proportion across different induction times. Quantification of the proportion of cells with active PG synthesis versus arrested PG synthesis at 0 h and 1 h after 50 ng/mL ATc induction corresponding to panel (B). Statistical significance of the difference in the proportion of synthesis-arrested cells between the two time points was determined by Pearson’s chi-square test.

****Supplementary Figure 6. Construction and characterization of the** E. coli **Δ*relA*Δ*spoT* background.**
A.** Semi-quantitative PCR confirming loss of relA and spoT amplicons in the constructed ΔrelAΔspoT strain; W3110 parental strain shows the expected bands. **B.** Representative radiolabeled TLC demonstrating substantially reduced (p)ppGpp accumulation in the ΔrelAΔspoT mutant compared with W3110. **C.** Representative HADA-labeled SIM images of the uninduced ΔrelAΔspoT strain and W3110 control, showing filamentation was observed in a subset of mutant cells, leading to an overall increase in bacterial chain length. **p* < 0.05, ***p* < 0.01, ****p* < 0.001.

****Supplementary Figure 7. IPTG-induced RelA expression drives (p)ppGpp accumulation, morphological remodeling and downregulation of conserved cell division-related genes in *Escherichia coli*.****

**A**. Thin-layer chromatography (TLC) analysis of intracellular (p)ppGpp levels in the *E. coli* Δ*relA*Δ*spoT* strain carrying the IPTG-inducible *relA* expression plasmid pQE80-*relA*. Cells were treated with 0 mM, 0.5 mM or 1 mM IPTG for 1 h, and intracellular ppGpp and pppGpp were detected by [γ-^32^P]-ATP-based radiolabeled TLC assay. **B**. Quantification of total intracellular (p)ppGpp levels corresponding to panel (A). Statistical significance between different IPTG concentration groups was determined by one-way ANOVA followed by Tukey’s multiple comparisons test. **C**. TLC analysis of intracellular (p)ppGpp levels in the *E. coli* Δ*relA*Δ*spoT*/pQE80-*relA* strain treated with 1 mM IPTG for 0 h, 0.5 h or 1 h. **D**. Quantification of total intracellular (p)ppGpp levels corresponding to panel (C). Statistical significance between different induction time groups was determined by one-way ANOVA followed by Tukey’s multiple comparisons test. **E**. Structured illumination microscopy (SIM) imaging of *E. coli* Δ*relA*Δ*spoT*/pQE80-*relA* cell morphology under different IPTG concentrations. Cells were treated with 0 mM, 0.5 mM or 1 mM IPTG for 1 h, and labeled with HADA to visualize cell morphology. Scale bar, 5 µm. **F**. Quantification of cell morphological parameters corresponding to panel (E). Quantification of cell length and cell width from the SIM images in panel (E). Individual data points (n ≥ 300 cells per condition) are shown with median and interquartile range indicated in violin plots. Statistical significance between different IPTG concentration groups was determined by one-way ANOVA followed by Tukey’s multiple comparisons test. **G**. SIM imaging of *E. coli* Δ*relA*Δ*spoT*/pQE80-*relA* cell morphology at different induction time points. Cells were treated with 1 mM IPTG for 0 h, 0.5 h or 1 h, and labeled with HADA to visualize cell morphology. Scale bar, 5 µm. **H**. Quantification of cell morphological parameters corresponding to panel (G). Quantification of cell length and cell width from the SIM images in panel (G). Individual data points (n ≥ 300 cells per condition) are shown with median and interquartile range indicated in violin plots. Statistical significance between different induction time groups was determined by one-way ANOVA followed by Tukey’s multiple comparisons test. **I**. Semi-quantitative PCR analysis of *ftsX* and *pbp3* transcription in *E. coli*. **p* < 0.05, ***p* < 0.01, ****p* < 0.001.
