# Supplementary material for: RelA-mediated (p)ppGpp accumulation links glucose starvation to peptidoglycan synthesis arrest and spherical morphogenesis via PBP2x and FtsX in Streptococcus suis
Source: J Bacteriol. 2026 Jul 14;208(8):e00030-26. doi: 10.1128/jb.00030-26 (PMC13492137; doi:10.1128/jb.00030-26)
Supplement: Supplementary file 2 [file jb.00030-26-s0002.docx]

### ****Table 1. Bacterial strains used in this study****

| **Strain** | **Relevant genotype or description** | **Source or reference** |
| --- | --- | --- |
| **S. suis** | | |
| SC19 | Wild-type clinical isolate | Lab stock |
| **Δ**relA**Δ**relQ | SC19 with deletion of relA and relQ | **This study** |
| **P_g_–**relA | SC19 with constitutive RelA overexpression (P_g_ promoter) | **This study** |
| **Δ**relA**Δ**relQ **+ ATc-**relA | ΔrelAΔrelQ carrying ATc-inducible RelA plasmid | **This study** |
| **Δ**ftsX | SC19 with deletion of ftsX | Lab stock |
| **E. coli** | | |
| DH5α | Cloning host | Lab stock |
| BL21(DE3) | Protein expression host | Lab stock |
| W3110 | Wild-type K-12 strain | Lab stock |
| **Δ**relA**Δ**spoT | W3110 with unmarked deletion of relA and spoT | **This study** |
| **Δ**relA**Δ**spoT **+ IPTG-**relA | ΔrelAΔspoT carrying IPTG-inducible RelA plasmid | **This study** |
| **S. pneumoniae** | | |
| D39 Δ*cps rpsL*^+^ | D39 Δ*cps rpsL*^+^*; Str^R^* | Lab stock |

### ****Table 2. Plasmids used in this study****

| **Plasmid** | **Key features** | **Source or reference** |
| --- | --- | --- |
| **pSET2-P_g_-*relA*** | Constitutive RelA expression in S. suis; Spc^R^ | **This study** |
| **ATc-inducible** relA **plasmid（**pSET2- P_tetR_-**RelA）** | ATc-regulated RelA expression in S. suis; Spc^R^ | **This study** |
| pET28a | Protein expression vector; Kan^R^ | Lab stock |
| **pET28a-**relA | For His-tagged RelA expression in E. coli | **This study** |
| **IPTG-inducible** relA **plasmid (pQE80-**relA**)** | IPTG-regulated RelA expression in E. coli; Amp^R^ | **This study** |
| pSET4s | Temperature-sensitive suicide vector for S. suis; Spc^R^ | Lab stock |
| pKD46 | λ-Red recombinase expression vector; Amp^R^ | Lab stock |
| pCP20 | FLP recombinase vector for marker excision; Amp^R^;Chl^R^ | Lab stock |

### ****Table 3. Oligonucleotide primers used in this study****

| **Primer name** | **Sequence (5′→3′)** | **Purpose** |
| --- | --- | --- |
| **For construction of Pg–**relA **strain** | | |
| P_g_-F | ggtacccggggatccTAAGATGAACCGGTA | Forward primer to amplify the constitutive promoter Pg (adds 5′ homology to vector). |
| P_g_-R | gttaatttctttcatGAATGATTTCCTCCTTATG | Reverse primer for Pg; uppercase letters denote gene-specific sequence, lowercase letters denote the overlap region with relA. |
| RelA-F | gaggaaatcattcATGAAAGAAATTAACTACACTGGGGA | Forward primer for relA; lowercase denotes the overlap region with P_g_, and uppercase contains the relA start codon. |
| RelA-R | gactctagaggatccTTATCCGTTGGTCCTT | Reverse primer to amplify relA (adds 3′ homology/restriction site). |
| **For construction of Δ**relA**Δ**relQ **mutant** | | |
| RelA-KO-uF | aaacgacggccagtgaattcTAAAAAATAACCGTGAACTC | Upstream homology for relA deletion |
| RelA-KO-uR | taagaggaagagtagagatgatGAAAATTGTATTACAAC |  |
| RelA-KO-dF | cacgttgtaatacaattttcatcATCTCTACTCTTCCTCTTATA | Downstream homology for relA deletion |
| RelA-KO-dR | gtcgactctagaggatccTGGTATAAGGGGTGTAGAGAAA |  |
| RelQ-KO-uF | ctgttgcAAAGTCCATACGGCCCCTTT | Upstream homology for relQ deletion |
| RelQ-KO-uR | caggtcgactctagaggatccCCGGATAGCTTGTTGATTGGA |  |
| RelQ-KO-dF | aaaacgacggccagtgaattcAGAGGATCCCTCTTCAGCAG | Downstream homology for relQ deletion |
| RelQ-KO-dR | ccgtatggactttGCAACAGTGATGATACAGATG |  |
| λ-Red-mediated gene knockout construction | | |
| **ec**RelA**-KO-F** | gtagatacagtatatatcaatctacattgtagatacgagcaaatttcggc  AAACGACGGCCAGTGAATTC | Primer with 5’ homology arm for relA deletion. Contains a 50-nt genomic homology (lowercase letters) and a 20-nt overhang (uppercase letters) for amplification of the resistance cassette. |
| **ec**RelA**-KO-R** | Tagttgcgatttgccgatttcggcaggtctggtccctaaaggagaggacg  ACCATGATTACGCCAAGCTT |  |
| **ec**SpoT**-KO-F** | ctggcgagcatttcgcagatgcgtgcataacgtgttgggttcataaaaca  AAACGACGGCCAGTGAATTC | Primer with 5’ homology arm for spoT deletion. Contains a 50-nt genomic homology (lowercase letters) and a 20-nt overhang (uppercase letters) for amplification of the resistance cassette. |
| **ec**SpoT**-KO-F** | Ccgttaccgctattgctgaaggtcgtcgttaatcacaaagcgggtcgccc  ACCATGATTACGCCAAGCTT |  |
| **For ATc-inducible** relA **plasmid** | | |
| tetR-F | agttaatttctttcatTATATTACTCTCCTTTGAGTTTAAAATTGTTA | Amplify tetR for ATc-inducible vector |
| tetR-R | tgcaggtcgactctagaggatccTTAAGACCCACTTTCACA |  |
| P_tetR_-RelA-F | taaaacgacggccagtgaattcTTATCCGTTGGTCCTTTTCA | Amplify relA for ATc-inducible vector |
| P_tetR_-RelA-R | taaactcaaaggagagtaatataATGAAAGAAATTAACTACACTGGGG |  |
| **For semi-quantitative PCR** | | |
| sspbp2x-RT-F | TGCAAACCAAGCACAGATGC | Detect pbp2x transcript in S. suis |
| sspbp2x-RT-R | TGACCATCTTCGCTGGCAAT |  |
| ssftsX-RT-F | GCAAAGCTCGCTTCGGATTT | Detect ftsX transcript in S. suis |
| ssftsX-RT-R | CAACTTCCCCATCCCGTACC |  |
| sspbp2b-RT-F | GGTAGTGCGCTTTACTCGGA | Detect pbp2b transcript in S. suis |
| sspbp2b-RT-R | AGGTCTGACCGAGTAGTCCC |  |
| ssftsW-RT-F | TTACAGATTGGCGGGTGGTC | Detect ftsW transcript in S. suis |
| ssftsW-RT-R | CCAAGCCATCCACCGTTACT |  |
| ssdivIVA-RT-F | TTTGCGAGAACGTTTGGCTT | Detect divIVA transcript in S. suis |
| ssdivIVA-RT-R | TCACGAAAGGCTTCGTCACT |  |
| ssftsE-RT-F | TGAAGCGGGAGAATTTGCCT | Detect ftsE transcript in S. suis |
| ssftsE-RT-R | CACGGGCAATAGCAATACGC |  |
| sspcsB-RT-F | CAGAATCGCAGACATTGGCG | Detect pcsB transcript in S. suis |
| sspcsB-RT-R | AGCAGCTTCCAATTCAGCCT |  |
| ssmltG-RT-F | ATCGCGGCAATCATTTGCTC | Detect *mltG* transcript in S. suis |
| ssmltG-RT-R | AGAAAGCGGAACTCCGACAG |  |
| ss16S-RT-F | GCCTCATAGCGGGGGATAAC | Internal control for S. suis |
| ss16S-RT-R | ACTCTCCCCTTCTGCACTCA |  |
| spnpbp2x-RT-F | CCTGCCAAACGTGGGACTAT | Detect pbp2x transcript in S. pneumoniae |
| spnpbp2x-RT-R | CCGTCTGTCCCTGCAAGA |  |
| spnFtsX-RT-F | AGATTTTTTCGCCATTTATTTG | Detect ftsX transcript in S. pneumoniae |
| spnFtsX-RT-R | GTATTGGCACCGCCATCTTGAA |  |
| spngapdh-RT-F | TTATGGTGCCCTCCAAACG | Internal control for S. pneumoniae |
| spngapdh-RT-R | GTCTATCAATCCAGCCTGGTC |  |
| ecpbp3-RT-F | ATGAAAGCAGCGGCGAAAA | Detect pbp*3* transcript in E. coli W3110 |
| ecpbp3-RT-R | GGCGAGACTCTTCACGCAGA |  |
| ecFtsX-RT-F | CGGCAGTTTGGCGGGCGTCTTG | Detect ftsX transcript in E. coli W3110 |
| ecFtsX-RT-R | GTATTGGCACCGCCATCTTGAA |  |
| ecgapA-RT-F | TCGCATTGTTTTCCGTGCTG | Internal control for E. coli W3110 |
| ecgapA-RT-R | CAGACGAACGGTCAGGTCAA |  |
| **For verification of Δ**relA**Δ**relQ **in** S. suis | | |
| ssRelA-check-F | ATTGGACCTCTCGGTCCGTA | Confirm relA deletion |
| ssRelA-check-R | TCGTGAACGGGAAGAGTTGG |  |
| ssRelQ-check-F | AGAACCGTCTCGTTGCCATT | Confirm relQ deletion |
| ssRelQ-check-R | CGCAAGGCTAATCGGCATTC |  |
| **For verification of Δ**relA**Δ**spoT **in** E. coli | | |
| ecRelA-check-F | TCGGGTTAGCGACGTAATCG | Confirm relA deletion |
| ecRelA-check-R | TGCCAAACTGCTGCATGAAC |  |
| ecSpoT-check-F | TAATGGGCAGATGTCCGTGG | Confirm spoT deletion |
| ecSpoT-check-R | CCGCTTGGCTGAACTTTGTC |  |
